# The key role of DLX6 in nasopharyngeal carcinoma: metastasis, angiogenesis and tumor immune mechanism

**DOI:** 10.3389/fimmu.2025.1522580

**Published:** 2025-03-03

**Authors:** Hanxuan Yang, Ximing Zeng, Xiaochuan Chen, Sunqin Cai, Yi Li, Wenqian Xu, Jinghua Lai, Sufang Qiu

**Affiliations:** ^1^ Department of Radiation Oncology, Clinical Oncology School of Fujian Medical University, Fujian Cancer Hospital, Fuzhou, China; ^2^ Fujian Provincial Key Laboratory of Translational Cancer Medicine, Fuzhou, China; ^3^ Fuzhou Hospital of Traditional Chinese Medicine Affiliated to Fujian University of Traditional Chinese Medicine, Fuzhou, China

**Keywords:** distal-less homeobox 6, NPC, proliferation, angiogenesis, tumor immunity

## Abstract

**Background:**

Precise management of gene expression is vital for the correct growth and operational efficiency of cells. Early-stage nasopharyngeal carcinoma (NPC) often presents asymptomatically, leading to delayed diagnosis, limited treatment options, and increased risk of recurrence and metastasis. This study investigates the role of DLX6 in NPC pathogenesis to enhance early screening and treatment.

**Methods and materials:**

Immunohistochemistry and RNA sequencing were utilized to investigate the relationship between DLX6 expression in NPC tumor samples and clinical pathological factors. Clinical data from Fujian Cancer Hospital, along with sequencing analysis from the GEO databases, were used for bioinformatics analysis. NPC cell lines with DLX6 knockdown were established, and the impact of DLX6 on the biological traits of NPC cells was assessed through wound healing, transwell, colony formation, and EdU assays. The expression of EMT-related proteins and the PI3K-AKT pathways was examined using western blot analysis.

**Results:**

Immunohistochemistry validated the association between DLX6 and NPC prognosis, whereas RNA sequencing illustrated its expression levels in tissues and cells. Functional assays such as wound healing, transwell, and colony formation revealed that DLX6 knockdown adversely affected NPC cell proliferation, invasion, and migration. Bioinformatics analysis revealed that DLX6 is involved in pathways related to cell cycle, DNA replication, and cancer progression. Immune infiltration analysis showed that DLX6 expression affects the immune landscape in NPC, correlating positively with DC and TH17 cells, and negatively with cytotoxic T cells and B cells. Low DLX6 expression was associated with higher levels of chemokines and better immunotherapy outcomes.

**Conclusion:**

Our study indicates that DLX6 is a novel prognostic biomarker and potential therapeutic target for NPC, playing a critical role in metastasis, angiogenesis, and tumor immunity.

## 1 Introduction

Nasopharyngeal carcinoma, also known as NPC, is classified as an epithelial tumor affecting the head and neck region and exhibits a distinct geographical distribution (1). The 2020 Global Cancer Statistics report reveals that approximately 75% of NPC cases are diagnosed in Southeast Asia and Southern China (2). This high prevalence of NPC in the southern region of China, particularly in Guangdong Province, led to its colloquial name “Guangdong Cancer” during the early 20th century (3). The annual incidence of NPC can reach alarming levels, ranging from 25 to 30 cases per 100,000 individuals. This disease predominantly affects the age group of young adults, typically between 35 and 55 years old, thereby exerting a profound impact on both society and families alike (4). The development of nonkeratotic NPC is primarily attributed to the presence of Epstein-Barr virus (EBV) infection. However, other factors such as alcohol consumption, salted food intake, smoking, environmental exposure, and lifestyle choices also contribute to the incidence of NPC, particularly in the case of keratinized NPC (5, 6). Histopathological examination using a light microscope enables the World Health Organization (WHO) to classify NPC into three distinct subtypes: keratinizing squamous cell carcinoma (Type I), non-keratinizing squamous cell carcinoma (Type II), with Type II further divided into undifferentiated or differentiated subtypes (7). Due to its notable radiosensitivity, concurrent chemoradiotherapy has emerged as the primary treatment approach for NPC (8). Early-stage NPC demonstrates a promising prognosis, boasting a 5-year survival rate spanning from 80% to 95%.On the other hand, late-stage NPC is associated with a comparatively lower survival rate, approximately ranging from 40% to 50% (9). Following the initial treatment, approximately 30% of patients diagnosed with NPC will experience relapse or disease progression. In cases where patients have inoperable recurrent or metastatic NPC, the standard first-line treatment involves the administration of platinum-based drugs in combination with gemcitabine chemotherapy. This therapeutic approach aims to prolong patient survival and is considered the established protocol for managing such cases (10). Nevertheless, the median overall survival (OS) for such patients is merely around 29 months, indicating a relatively brief duration of survival (11). Because early-stage NPC is asymptomatic, a significant number of patients are diagnosed at an advanced stage of the disease. This delayed diagnosis restricts the available treatment options and increases the likelihood of disease recurrence and the development of distant metastasis (12). Hence, conducting comprehensive research on the mechanisms underlying the occurrence and progression of NPC holds immense importance. Such studies aim to diminish the rates of recurrence and metastasis, thereby enhancing both the survival rates and quality of life for individuals at advanced stages of the disease.

Distal-less homeobox 6 (DLX6) belongs to the NK homeobox gene family (13) and is primarily expressed in the first pharyngeal arch, brain, and skeletal tissue (14). It has been reported that DLX6 plays a significant role in the differentiation of chondrocytes and osteoblasts (15). Additionally, DLX6 is involved in the development of craniofacial structures, the inner ear, limbs, and the brain, particularly in the normal morphological development of the mandible (16, 17). However, the pathological and physiological functions of DLX6 remain poorly understood. Existing studies on DLX6 in tumors include the following: Liang, J. et al. analyzed DLX6 expression in a cohort of OSCC patients, with RNA sequencing data derived from 29 primary oral tumors and matched normal mucosal samples. The results demonstrated significantly higher DLX6 expression in oral cancer tissues compared to normal mucosal tissues (18). Diana Bell et al., through whole-genome sequencing of 42 cases of primary salivary gland adenoid cystic carcinoma and 5 normal salivary gland samples, identified DLX6 as a potential driver gene of salivary gland adenoid cystic carcinoma, showing high expression across various types of adenoid cystic carcinoma (19). Wei Yu et al. found that DLX6 was overexpressed in multiple glioma cell lines. Functional assays, CCK-8 experiments, and tumor formation experiments in BALB/c nude mice confirmed that DLX6 is a key gene in pathways enhancing intracellular autophagy, promoting tumor cell proliferation, and inhibiting tumor cell apoptosis (20). Stefan Nagel et al. confirmed the oncogenic role of DLX6 in lymphoid and myeloid malignancies through transcriptome analysis of gene expression profiles and RNA sequencing, alongside polymerase chain reaction (PCR) and Western blot analyses (21). Li Zhang et al., using RNA sequencing, analyzed postoperative pathological specimens from patients with hepatocellular carcinoma (HCC). The results showed that DLX6 and its antisense RNA, DLX6-AS1, were highly expressed in the more invasive/extensive S1 subtype of HCC. The strong correlation between DLX6-AS1 and DLX6 suggested that DLX6-AS1 may promote DLX6 transcription. This study highlighted that DLX6 and its antisense RNA, DLX6-AS1, could serve as prognostic markers for hepatocellular carcinoma (22).

## 2 Materials and methods

### 2.1 Patients and cell line

A total of 213 tissue samples were gathered, comprising 19 normal tissue samples and 194 NPC tissue samples, from Fujian Provincial Cancer Hospital (refer to **Supplementary Tables 1, 2**). All tissue specimens were immediately preserved in liquid nitrogen within 30 minutes. Additionally, we acquired ten NPC tissue samples, which included five frozen metastatic tumor biopsies and five non-metastatic tumor biopsies, from the Fujian Cancer Hospital between January and March 2015. Tissue microarray chips detailed in **Supplementary Table 3** were utilized for further analysis, which included 17 normal tissue samples and 103 NPC tissue samples. The human NPC cell lines HK-1 and CNE-2 were cultured in Roswell Park Memorial Institute-1640 (RPMI-1640) medium supplemented with 10% fetal bovine serum (FBS). All protocols were executed in accordance with the pertinent guidelines and regulations, and written informed consent was acquired from all researchers.

### 2.2 Database

Data was sourced from the GSE102349 dataset available in the GEO database (https://www.ncbi.nlm.nih.gov/), which includes RNA sequencing data and clinical characteristics of 113 NPC patients. Based on the median RNA sequencing expression levels, patients were categorized into two groups: DLX6-high and DLX6-low.

### 2.3 Survival curves

To highlight differences in survival duration, Kaplan-Meier survival curves were constructed, and the “survival” R package was used to determine statistical significance via log-rank p-values. Visual representations were generated using the website (https://www.xiantao.love/).

### 2.4 Immunohistochemistry staining

To evaluate the intensity of immunohistochemical (IHC) staining, a semiquantitative method was applied to 103 NPC tissues and 17 normal nasopharyngeal tissues(including 5 metastasis and 5 non-metastasis), all of which were fixed in formalin and embedded in paraffin. The tissue sections were stained with the relevant antibodies, as detailed in **Supplementary Table 3**. The stained samples were then examined under a microscope (3DHISTECH, Hungary) at 20× magnification, and images were captured for analysis. The histochemistry score (H-score) was used to quantify protein expression levels, calculated as follows: H-score = (percentage of cells with low staining intensity × 1) + (percentage of cells with moderate staining intensity × 2) + (percentage of cells with strong staining intensity × 3).

### 2.5 Plasmid constructs

A small interfering RNA (shRNA) targeting DLX6 was designed, and a lentivirus expressing this shRNA was created following a protocol described in a previous study (23). The shRNA was constructed with the target sequence (TACTCTGAAAGCAAGCAAGAA), while a scramble sequence (TTCTCCGAACGTGTCACGT) served as a negative control (NC) for the lentiviral experiment. Hairpin DNA oligonucleotides designed for the shRNA sequences were synthesized, annealed, and then cloned into the GV115 lentiviral vector, which contains the green fluorescent protein (GFP) gene. Lentiviral expression systems from Obio (China) were utilized to generate lentiviruses encoding either DLX6 shRNA or scrambled shRNA, adhering to standard procedures. The lentiviruses were separately infected into HK-1 and CNE2 cells and we screened for stably infected cells with puromycin. Western blotting techniques were employed to assess the efficiency of DLX6 knockdown.

### 2.6 Proliferation, migration and invasion

#### 2.6.1 5-Ethynyl-2′-Deoxyuridine analysis of cell proliferation

Cell proliferation in HK-1 and CNE2 cells was evaluated using an EdU kit from Beyotime (China). The two cell lines were treated with EdU at a concentration of 50 μmol/L, diluted 1:1000, for 2 hours. Following the incubation, the cells were fixed with 4% formaldehyde at 37°C for 15 minutes and then permeabilized with 0.5% Triton X-100 at the same temperature. The EdU-labeled cells were subsequently examined under a fluorescence microscope.

#### 2.6.2 Transwell assay

In serum-free medium, 1×10^5 cells were suspended and loaded onto upper transwell migration chambers from Corning Costar in California, USA. For the transwell invasion assay, Matrigel (BD, California, USA) was used to coat the chambers. A medium containing 10% FBS was added to the lower chambers. After a 24-hour incubation period, the membranes were treated with methanol for fixation and subsequently stained with 1% crystal violet.

#### 2.6.3 Wound healing assay

Following infections with either shDLX6 or shCtrl, HK-1 and CNE2 cells were analyzed against their respective controls. A straight scratch was made across the cell monolayer using a sterile pipette tip. After 24 or 48 hours, images of the migrating cells were captured, and the wound width was measured to assess the migration capacity of the cells.

#### 2.6.4 Colony formation assay

HK-1 and CNE2 cells were transfected with shDLX6 or shCtrl, then plated at 2500 cells per well in 6-well plates. The plates were kept in incubation for 14 days or until distinct colonies became visible. A Subsequently, the cells were treated with paraformaldehyde (Sangon Biotech Co., Ltd, Shanghai, China) for half an hour and stained with Crystal Violet (Beyotime, Shanghai, China) for a duration exceeding 20 minutes. The colonies were counted using a microscope for further analysis.

### 2.7 Western blotting

C Cells were lysed using RIPA Lysis Buffer (Abcam, USA) supplemented with 1% PMSF. The proteins were then separated by SDS-PAGE and transferred onto a PVDF membrane provided by Millipore Corp (USA). The membrane was blocked with QuickBlock™ Blocking Buffer for Western Blot (Beyotime, China) and subsequently incubated overnight at 4°C with primary antibodies targeting DLX6 (Proteintech, China), Snail (Proteintech, China), p-AKT (Cell Signaling Technology, USA), β-Catenin (Cell Signaling Technology, USA), N-cadherin (Cell Signaling Technology, USA), and β-Tubulin (Cell Signaling Technology, USA).

### 2.8 ssGSEA immune infiltration analysis

The RNA sequencing data from NPC patients were analyzed with the single-sample Gene Set Enrichment Analysis (ssGSEA) method. This approach facilitated the evaluation of immune cell infiltration by employing a gene set made up of markers specific to various immune cells, thereby allowing the quantification of the relative abundance of 22 different immune cell types within the NPC samples. To investigate the relationship between DLX6 expression levels and the abundance of immune cells, a Spearman rank correlation test was conducted.

### 2.9 Functional enrichment analysis

Patients were sorted into high and low DLX6 expression groups. A study of signaling pathways involved obtaining gene sets from MsigDB. Using GSEA(Gene Set Enrichment Analysis), enriched pathway gene sets were identified. These sets were then ranked by consistency scores.

### 2.10 Statistical analysis

Survival curves were constructed utilizing the Kaplan-Meier method, and distinctions were assessed via log-rank analysis. The relationship between DLX6 expression and immunotherapeutic response in NPC was examined using a chi-square test with continuity correction. Spearman’s rank correlation was used to evaluate the link between DLX6 expression and immune cell infiltration levels. Significance was established at a p-value below 0.05, with all statistical analyses conducted utilizing SPSS software (version 26.0) and R software (version 4.1.1).

## 3 Results

### 3.1 DLX6 is upregulated in NPC metastasis and correlates with an unfavorable prognosis

**Figure 1:**
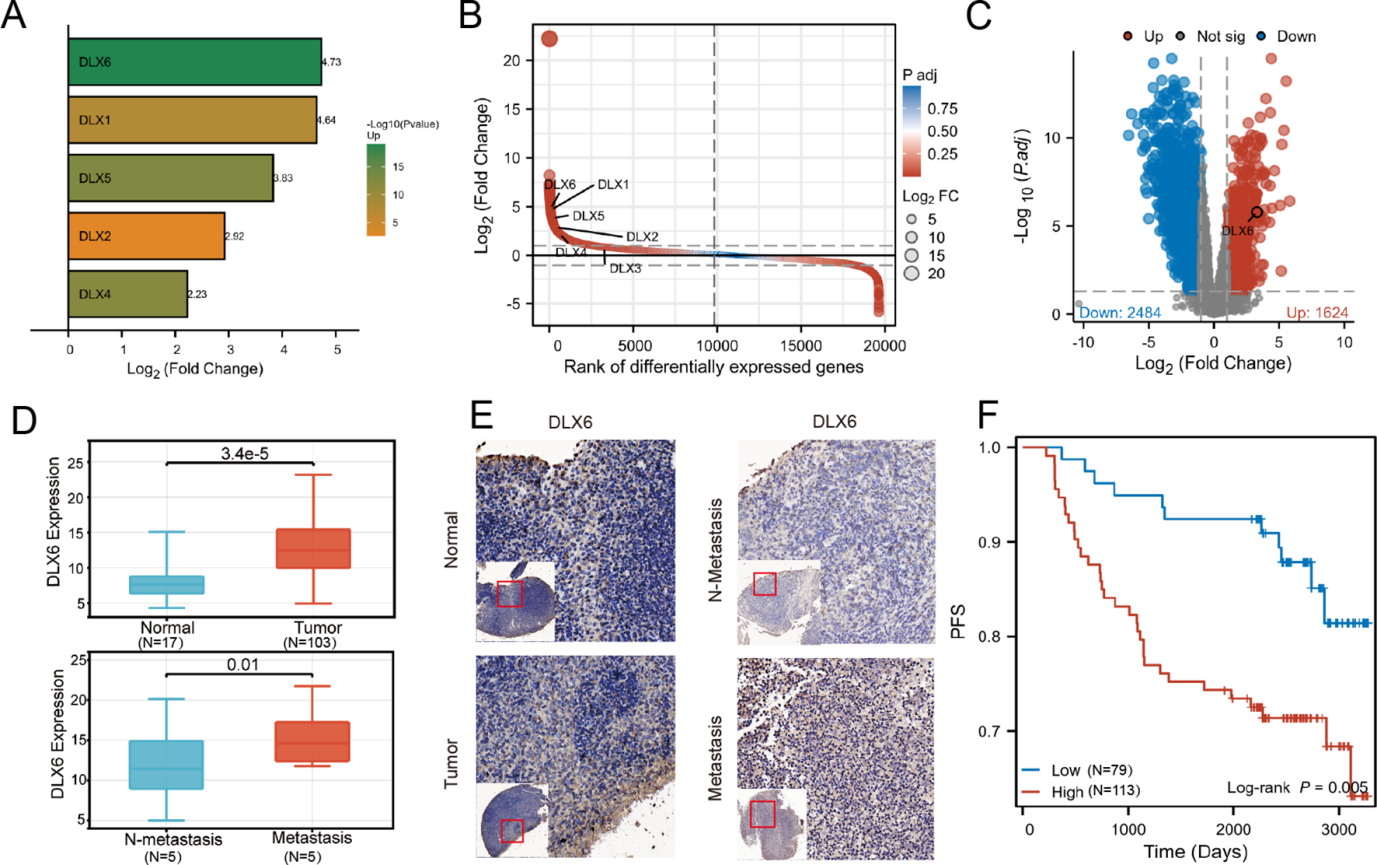
DLX6 is overexpressed in NPC samples and is associated with a poor prognosis. **(A)** Gene expression differences between cancer and adjacent tissues: DLX6 exhibits the largest difference within the DLX family. **(B)** Gene expression differences between cancer and adjacent tissues: DLX6 ranks among the top in nasopharyngeal cancer sequencing data. **(C)** Volcano plot shows DLX6 is highly expressed in NPC. **(D, E)** Immunohistochemical staining showed that DLX6 expression was higher in patients with NPC and NPC metastasis groups. **(F)** DLX6 patients with high expression of DLX6 protein had a significantly worse progression-free survival (PFS).

Considering the important role of the DLX family in the development of the head and face, we hope to further explore the relationship between the DLX family and the development of NPC. We analyzed the sequencing and clinical data of patients in Fujian Cancer Hospital and observed a significant elevation in DLX6 expression in nasopharyngeal tissues compared to para-cancerous tissues, which was the most obvious difference in expression between NPC and paracancerous tissues in the DLX family (**Figures 1A–C**). Further, mRNA expression profiles of five metastatic and five non-metastatic NPC tissues were analyzed to determine whether DLX6 played a role in NPC metastasis. The expression levels of DLX6, as revealed by immunohistochemical staining of NPC samples and normal nasopharyngeal specimens, confirmed that DLX6 expression was significantly elevated not only in NPC compared to normal nasopharyngeal specimens but also in NPC metastasis. (**Figures 1D–E**). The Kaplan-Meier survival plot also illustrated that patients with higher DLX6 expression experienced a significantly worse prognosis (p = 0.005) (**Figure 1F**). And it had also been validated in the GEO database (**Supplementary Figure 1A**).

### 3.2 Functional enrichment analysis

**Figure 2:**
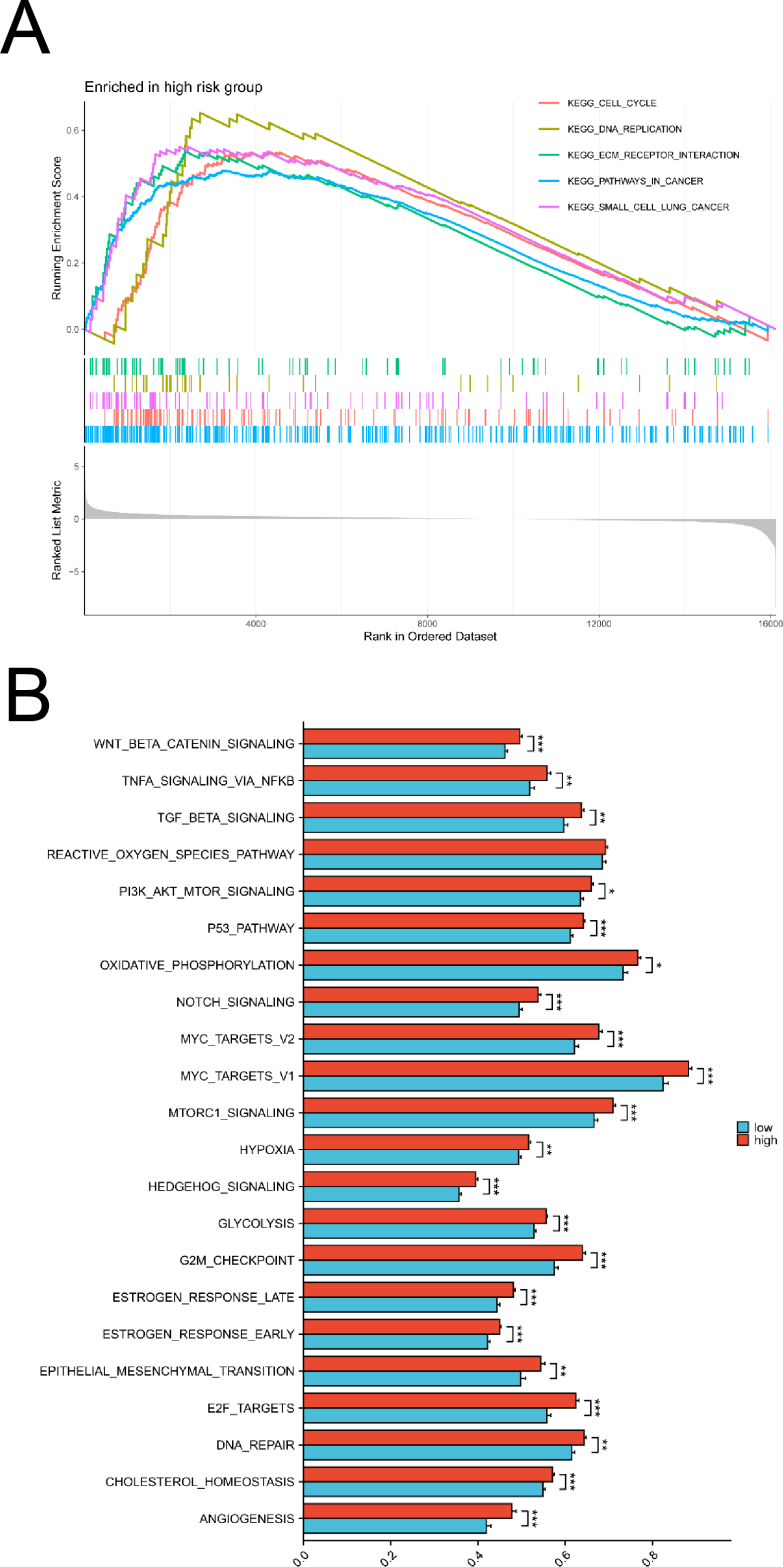
Functional enrichment analysis of DLX6. **(A)** KEGG analysis between high and low DLX6 expression groups. **(B)** The functional enrichment of DLX6 expression groups was assessed through ssGSEA using sequencing data from patients at Fujian Provincial Cancer Hospital. *p<0.05, **p<0.01, ***p<0.001.

Through GSEA KEGG enrichment analysis using sequencing and clinical data from patients at Fujian Cancer Hospital, we found that DLX6 was significantly enriched in t cell cycle, DNA replication, ECM receptor reaction and pathways in cancer (**Figure 2A**). We further conducted an ssGSEA analysis of cancer hallmarks using sequencing and clinical data from patients at Fujian Cancer Hospital to elucidate the potential pathways and biological processes associated with DLX6. Our analysis revealed that several pathways were enriched in the high DLX6 expression group, including angiogenesis, cholesterol homeostasis, DNA repair, E2F targets, epithelial-mesenchymal transition, early and late estrogen response, G2M checkpoint, glycolysis, hedgehog signaling, hypoxia, mTORC1 signaling, Myc targets V1 and V2, Notch signaling, oxidative phosphorylation, the p53 pathway, PI3K/Akt/mTOR signaling, the reactive oxygen species pathway, TGF-beta signaling, TNF-alpha signaling via NF-kB, and Wnt/beta-catenin signaling. (**Figure 2B**). Most of the enriched pathways have also been validated in the GEO database (**Supplementary Figure 1B**).

### 3.3 DLX6-shRNA inhibited NPC proliferation, migration and invasion

**Figure 3:**
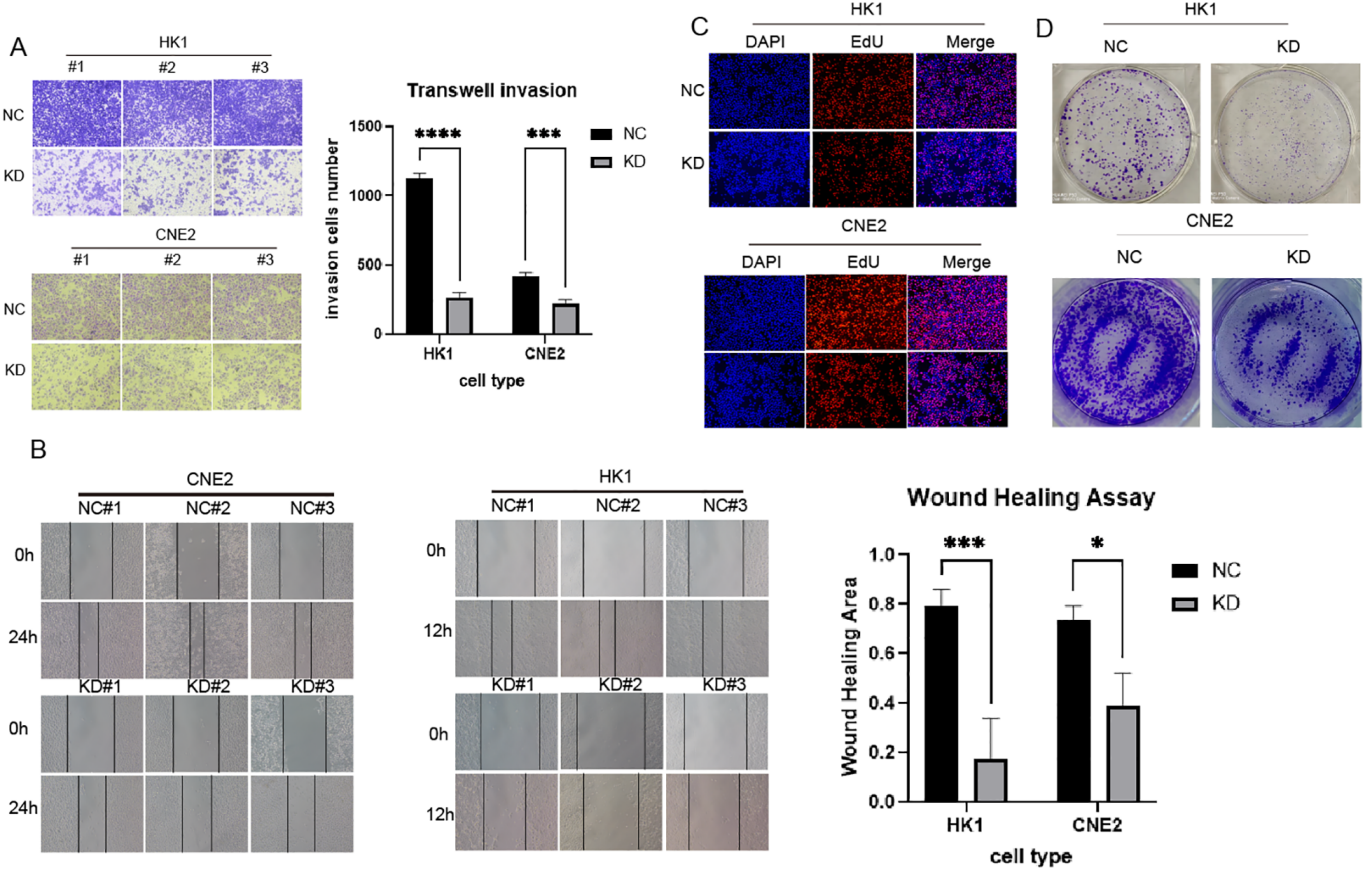
DLX6-shRNA inhibited NPC proliferation, migration and invasion. **(A)** Transwell assays were performed to indicate the invasion of DLX6 knockdown HK-1 and CNE2 cells compared to the nc group.**(B)** Migration ability of DLX6 knockdown HK-1 and CNE2 cells compared to the nc group by wound healing test. **(C, D)** The 5-ethynyl-2′-deoxyuridine (EdU) analysis **(C)** and Colony formation **(D)** was used to detect the proliferation of DLX6 knockdown HK-1 and CNE2 cells compared to the nc group. *p<0.05, ***p<0.001,****p<0.0001.

To downregulate DLX6 expression, HK-1 and CNE2 cell lines were transfected with shDLX6, and the effectiveness of these infections was verified using Western blotting. The influence of DLX6 on the migratory and invasive characteristics of NPC cells through a Transwell assay. This analysis indicated a significant decline in cell migration and invasion following DLX6 downregulation (**Figure 3A**). Moreover, the wound healing assay showed that shDLX6 cells had a substantially lower migration rate compared to shCtrl cells (**Figure 3B**). The 5-ethynyl-2′-deoxyuridine (EdU) assay (**Figure 3C**) subsequently demonstrated that reduced DLX6 expression significantly decreased the proliferative capacity of NPC cells in comparison to the control group. During the colony formation assay, a significant decrease in the colony count was observed in the shDLX6 group compared to the shCtrl group (**Figure 3D**).

**Figure 4:**
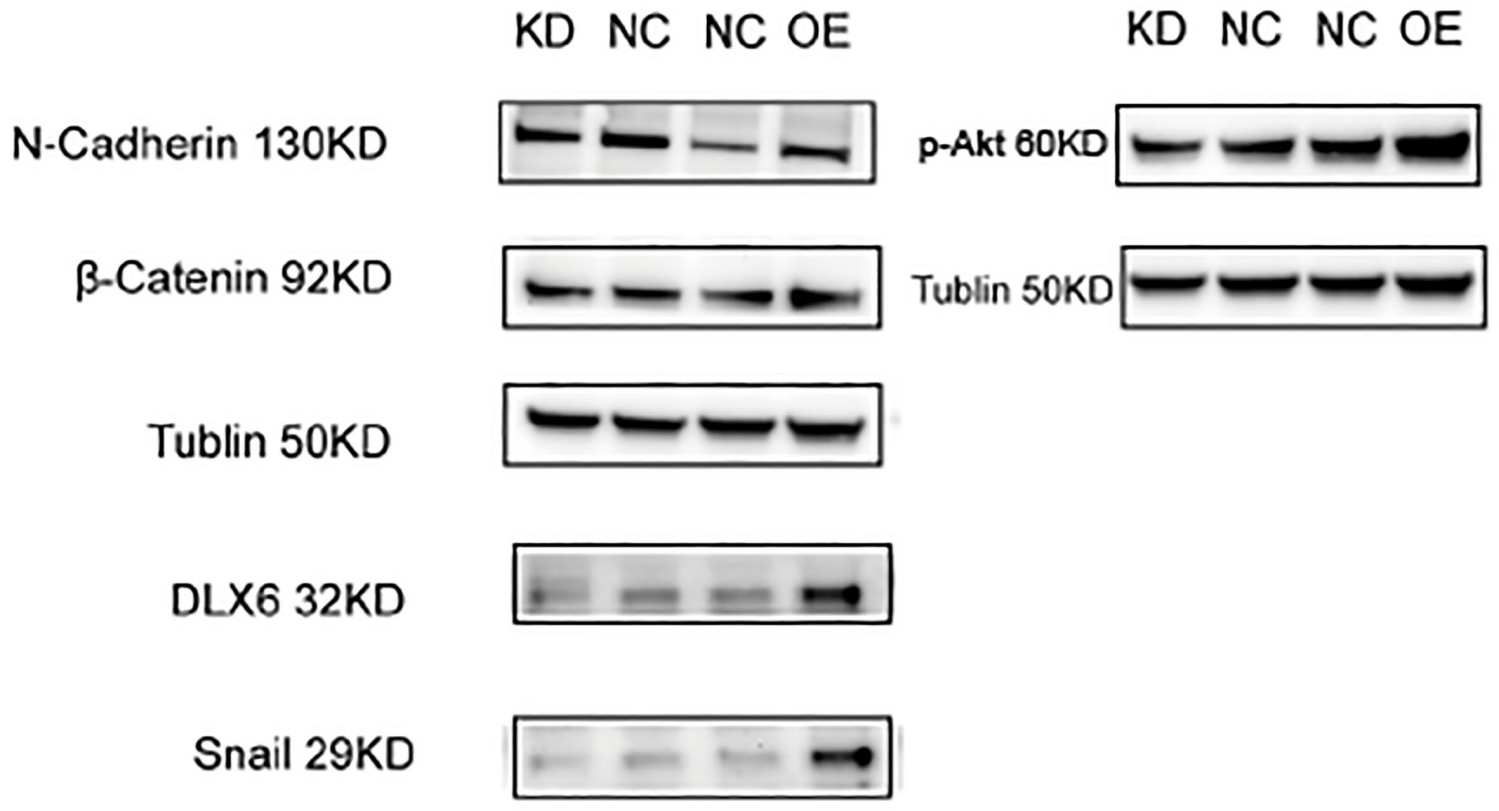
DLX6 modulates NPC metastasis via the PI3K/AKT signaling. Western blotting showed a decreased expression of N-Cadherin, β-catenin, snail and p-AKT after DLX6 Knockdown, and an increased expression of N-Cadherin, β-catenin, snail and p-AKT after DLX6 overexpression.

The effect of DLX6 on the levels and activation states of proteins involved in metastasis-related signaling pathways was evaluated using Western blot analysis. In HK-1 cells, knocking down DLX6 resulted in reduced levels of p-AKT. Further examination of metastasis-related downstream proteins in the PI3K/AKT pathway showed that DLX6 knockdown led to decreased expression of Snail, β-Catenin and N-cadherin. At the same time, in HK-1 cells, overexpression of DLX6 resulted in increased levels of p-AKT. Further examination of metastasis-related downstream proteins in the PI3K/AKT pathway revealed that DLX6 overexpression resulted in increased expression of Snail, β-Catenin, and N-cadherin (**Figure 4**).

### 3.4 Immune infiltrations analysis

**Figure 5:**
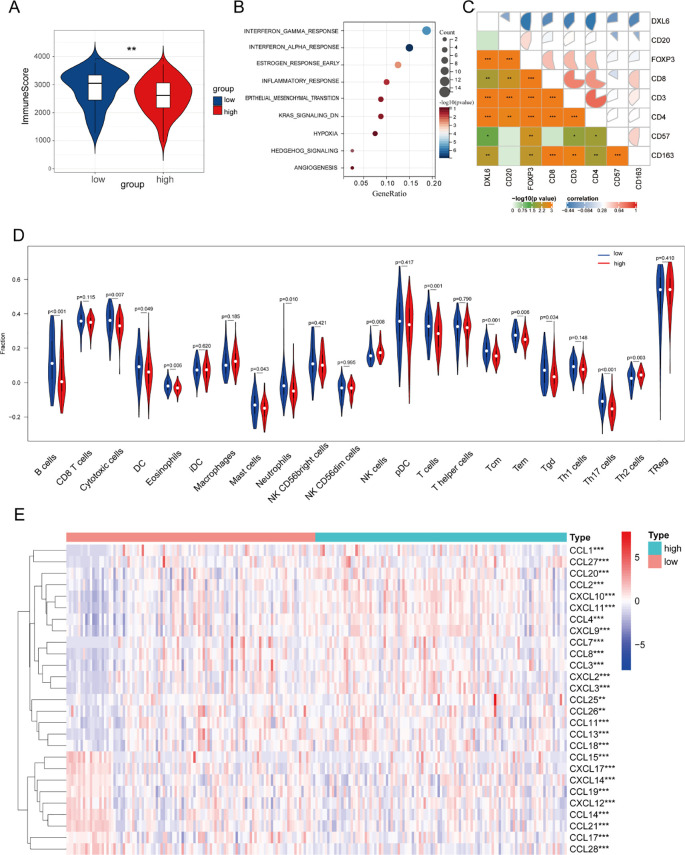
Immune infiltration analysis. **(A)** The relationship between DLX6 expression and immune score. **(B)**The functional enrichment of DLX6 expression groups was assessed through ssGSEA using sequencing data from ctrl-DLX6 and sh-DLX6 HK-1 cells. **(C)** DLX6 protein expression exhibited a negative correlation with immune cell markers. **(D)** DLX6 expression was found correlated with immune cell types via functional enrichment analysis using ssGSEA. **(E)** Immune-related chemokines and chemokine receptors were studied in relation to DLX6 expression. The sequencing data of figure **(A,C-E)** is from patient samples at Fujian Provincial Cancer Hospital. *p<0.05, **p<0.01, ***p<0.001.

To investigate the relationship between DLX6 and tumor immunity, we initially examined the correlation between DLX6 expression and the Immunescore. The findings indicated that high DLX6 expression was associated with a significant reduction in the immunity score, suggesting that DLX6 may play an inhibitory role in tumor immunity (**Figure 5A**). Furthermore, sequencing analysis of ctrl-DLX6 and sh-DLX6 HK-1 cells revealed that DLX6 was significantly enriched in the INF-γ and INF-α pathways (**Figure 5B**).

Following this, we investigated the association between the infiltration of immune cells and the expression of DLX6 protein within NPC samples sourced from our database. Our results revealed that different levels of DLX6 expression were associated with distinct immune landscapes, as depicted in a box plot (**Figure 5D**). High expression of DLX6 may promote tumor growth and metastasis by inhibiting the infiltration of various immune cells, thereby weakening tumor immune surveillance. Specifically, the high expression of DLX6 suppresses the infiltration of B cells, cytotoxic cells, T cells, neutrophils, central memory T cells, effector memory T cells, γδT cells, and dendritic cells, all of which play key roles in tumor immune responses, including antitumor cytotoxicity, immune memory formation, and the initiation of specific immune responses. By reducing the infiltration of these immune cells, tumors may escape immune surveillance, promoting their growth and metastasis. Beyond the transcriptional findings, IHC staining further confirmed the negative association between DLX6 and the expression of CD20, CD3, CD4, CD8, CD57, CD163, and FOXP3 at the protein level (**Supplementary Tables 4, 5**; **Figure 5C**).

In the final step, we examined the relationship between DLX6 expression and the expression of immune-related chemokines and their receptors. Chemokines are vital for immune system development and maintenance, and they play a major role in regulating immune and inflammatory responses. Targeting specific chemokines within solid tumors holds potential for enhancing cancer immunotherapy by altering the immune landscape within the tumor microenvironment and has emerged as a new checkpoint in cancer therapy (24–27). Our findings demonstrate a strong correlation between DLX6 expression and immune-related chemokines and their receptors, highlighting DLX6’s critical role in tumor immunity and its potential as a target for immunotherapy (**Figure 5E**).

### 3.5 Immunotherapy related analysis

**Figure 6:**
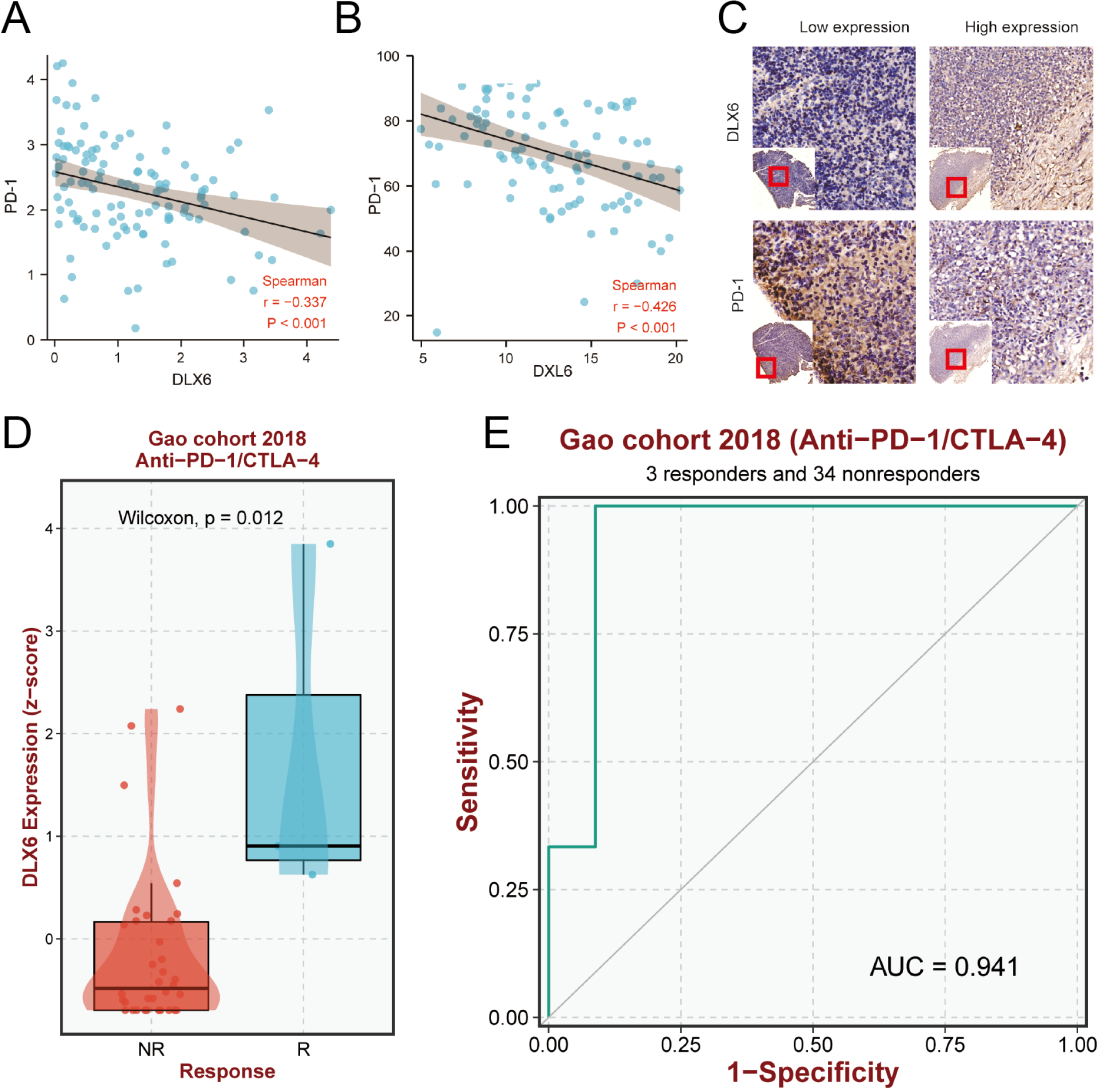
Prediction of immunotherapy efficacy. **(A)** Scatterplots of the correlations visualizing the mRNA expression of DLX6 with PD-1. **(B)** Scatterplots of the correlations visualizing the protein expression of DLX6 with PD-1 via immunohistochemistry analysis. **(C)** Immunohistochemistry analysis of the PD-1 protein expression in NPC tissues with low and high DLX6 expression. **(D, E)** The relationship between DLX6 expression and immunotherapy response **(D)** and sensitivity ROC **(E)** of (Anti-PD-1/CTAL-4).

Considering the role of DLX6 in tumor immunity, we analyzed the potential impact of DLX6 in immunotherapy. Through GEO and our database, we observed the negative correlation between DLX6 and PD-1 expression in the GSE102349 dataset (p < 0.001, r = -0.377; **Figure 6A**). Meanwhile, in addition to the findings based on the transcription levels, a decrease in PD-1 levels was observed in conjunction with the increase in DLX6 protein expression via IHC from our database (p < 0.001, r = −0426; **Figure 6B**). The same conclusion was also evident in the immunohistochemical analysis (**Figure 6C**). Moreover, our analysis delved into the influence of DLX6 on immunotherapy, revealing a substantial effect of DLX6 on both the prognosis and efficacy of immunotherapy. In Gao cohort 2018, high DLX6 expression may improve the response of Anti-PD1/CTA4 therapy (**Figure 6D**), and may also improve the sensitivity of Anti-PD1/CTA4 therapy (**Figure 6E**).

## 4 Discussion

NPC is a malignancy predominantly affecting populations in Southeast Asia and Southern China (28). The disease is often associated with Epstein-Barr virus (EBV) infection and exhibits a high propensity for metastasis, contributing to poor prognosis and treatment challenges (29). Current therapeutic strategies for NPC include radiotherapy, chemotherapy, and emerging immunotherapies (30). Despite these advancements, the prognosis for metastatic NPC remains poor, necessitating the identification of novel molecular targets for therapeutic intervention (31).

DLX6, a member of the distal-less homeobox (DLX) gene family, has garnered attention in cancer research due to its role in developmental processes and tumor progression. Elevated DLX6 expression has been implicated in several cancers, including hepatocellular carcinoma and colorectal cancer, where it promotes proliferation, invasion, and metastasis (18–22). Meanwhile, studies have shown that its antisense RNA, DLX6-AS1, can promote the malignant phenotype of NPC (32). However, the specific role of DLX6 in NPC metastasis and prognosis had not been comprehensively studied until now.

Our research scrutinized sequencing data and clinical records of patients at Fujian Cancer Hospital to explore the expression and function of DLX6 in NPC. The findings indicated a notable elevation in DLX6 expression levels within nasopharyngeal tissues in contrast to para-cancerous tissues. This elevated expression was further confirmed through mRNA expression profiles and immunohistochemical staining, showing a marked increase in DLX6 levels in metastatic NPC tissues compared to non-metastatic tissues. Kaplan-Meier survival analysis demonstrated that high DLX6 expression correlated with significantly worse prognosis.

Functional assays performed on NPC cell lines (HK-1 and CNE2) with DLX6 shRNA demonstrated that the knockdown of DLX6 effectively reduced its expression, resulting in a marked decrease in the proliferation, migration, and invasion abilities of the cells. These findings were further supported by EDU, colony formation, Transwell, and wound healing assays. Additionally, ssGSEA analysis revealed several pathways associated with elevated DLX6 expression, including angiogenesis, epithelial-mesenchymal transition (EMT), and key signaling pathways such as PI3K/AKT and Wnt/β-catenin, which was validated by Western blot (WB) analysis. The Western blot results for DLX6-knockdown cells showed a reduction in p-AKT levels, a key element of the PI3K/AKT signaling pathway. This was accompanied by decreased Snail, β-catenin and N-cadherin expression. And at the same time, an increased expression of N-Cadherin, β-catenin, snail after DLX6 overexpression, suggesting a reversal of EMT.

Immune infiltration analysis revealed a complex relationship between DLX6 expression and the immune landscape in NPC samples. High expression of DLX6 may promote tumor growth and metastasis by inhibiting the infiltration of various immune cells, thereby weakening tumor immune surveillance. Specifically, the high expression of DLX6 suppresses the infiltration of B cells, cytotoxic cells, T cells, neutrophils, central memory T cells, effector memory T cells, γδT cells, and dendritic cells, all of which play key roles in tumor immune responses, including antitumor cytotoxicity, immune memory formation, and the initiation of specific immune responses. By reducing the infiltration of these immune cells, tumors may escape immune surveillance, promoting their growth and metastasis. Further exploration of DLX6’s involvement in immunotherapy response suggested that reduced DLX6 expression correlated with elevated levels of chemokines responsible for attracting immune cells. The DLX6-low group demonstrated higher expression levels of Immune-related chemokines and chemokine receptors, which are indicative of a favorable response to anti-PD-1 therapies. And really had a better immunotherapy outcome in the Gao cohort 2018.

In this study, we identified the significant role of DLX6 in NPC, particularly its potential in cancer metastasis, angiogenesis, and tumor immune mechanisms. However, several limitations of the study need further exploration. First, the sample size was relatively small, and the data were obtained from a single institution, which may limit the generalizability of the results. Although both cell line models and clinical samples were used, the reliance on *in vitro* models may not fully capture the complexities of human tumors, especially regarding tissue heterogeneity and the immune microenvironment. Additionally, while shRNA technology is commonly used for gene function studies, it may have off-target effects, potentially affecting the accuracy of the results. The clinical data were cross-sectional, and although a correlation between DLX6 expression and NPC prognosis was observed, causality cannot be established. Finally, while we identified several key signaling pathways associated with DLX6, the specific molecular mechanisms by which DLX6 regulates these pathways remain unclear. Future studies should validate these findings through larger, multi-center studies, *in vivo* models, and gene editing techniques such as CRISPR-Cas9, and further explore the role of DLX6 in NPC.

However, in conclusion, the study revealed that DLX6 plays a significant role in NPC progression and metastasis. Elevated DLX6 expression is associated with a poor prognosis, increased proliferative, migratory, and invasive tendencies of NPC cells, and altered immune landscapes that may impact the effectiveness of immunotherapies. By addressing these limitations and pursuing these future research directions, we can gain a deeper understanding of DLX6’s role in NPC and its potential as a therapeutic target, ultimately contributing to more effective treatments and improved patient outcomes.

## Data Availability

The datasets presented in this study can be found in online repositories. The names of the repository/repositories and accession number(s) can be found in the article.

## Supplementary material

Supplementary Figure 1
**(A)**DLX6 patients with high expression of DLX6 protein had a significantly worse progression-free survival (PFS) in GEO database.**(B)**The functional enrichment of DLX6 expression groups was assessed through ssGSEA using sequencing data from GEO database.

Supplementary Figure 2 Original image of Western-blot.
