# Supplementary figures and images for: The key role of DLX6 in nasopharyngeal carcinoma: metastasis, angiogenesis and tumor immune mechanism
Source: Front Immunol. 2025 Mar 3;16:1522580. doi: 10.3389/fimmu.2025.1522580 (PMC11911523; doi:10.3389/fimmu.2025.1522580)

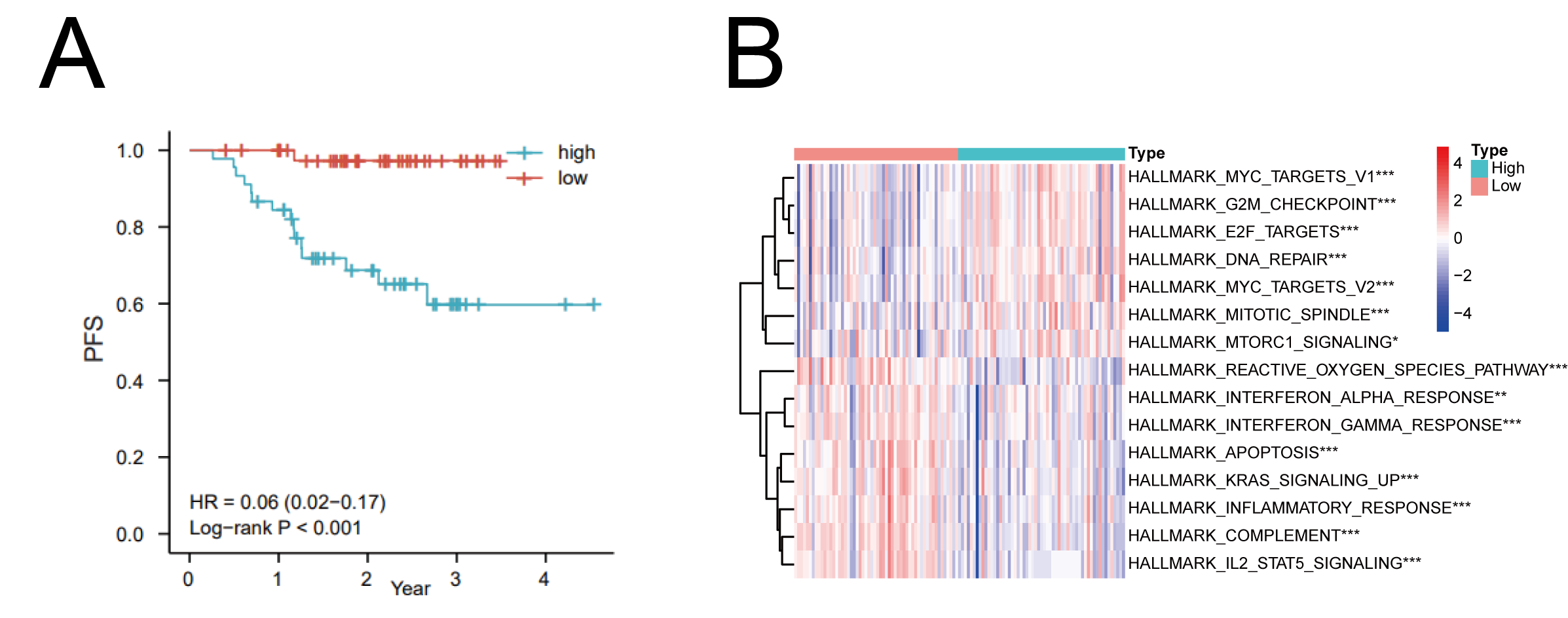

Supplement: Supplementary Figure 1 — (A)DLX6 patients with high expression of DLX6 protein had a significantly worse progression-free survival (PFS) in GEO database.(B)The functional enrichment of DLX6 expression groups was assessed through ssGSEA using sequencing data from GEO database. [file Image1.tif]

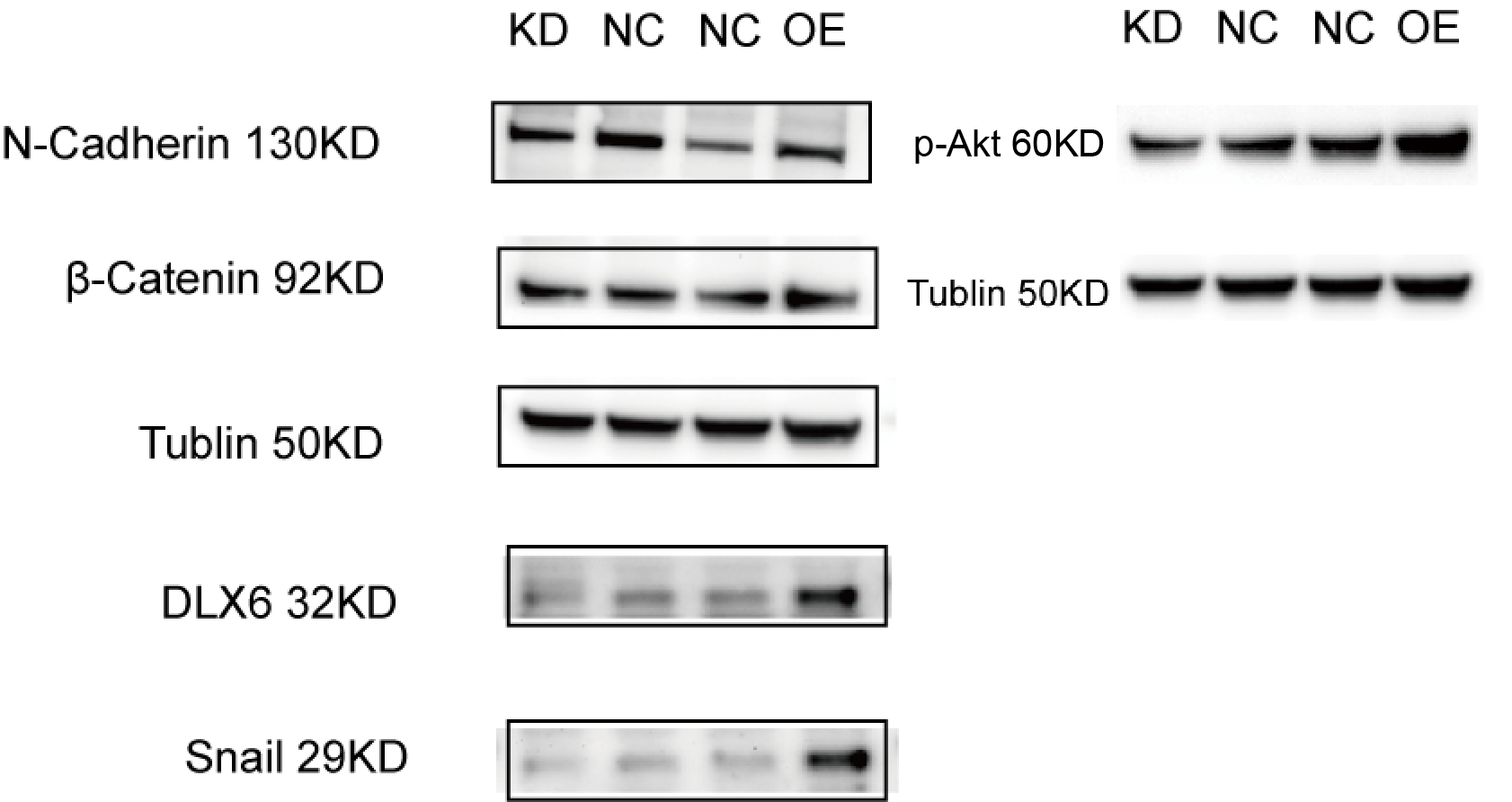

Supplement: Supplementary Figure 2 — Original image of Western-blot. [file Image2.tif]
